# Label-Free Capacitive Biosensor for Detection of *Cryptosporidium*

**DOI:** 10.3390/s19020258

**Published:** 2019-01-10

**Authors:** George Luka, Ehsan Samiei, Soroush Dehghani, Thomas Johnson, Homayoun Najjaran, Mina Hoorfar

**Affiliations:** 1 School of Engineering, University of British Columbia, 333 University Way, Kelowna, BC V1V 1V7, Canada; george.luka@alumni.ubc.ca (G.L.); soroushd@ece.ubc.ca (S.D.); thomas.johnson@ubc.ca (T.J.); homayoun.najjaran@ubc.ca (H.N.); 2 Department of Mechanical & Industrial Engineering, University of Toronto, Toronto, ON M5S 3G8, Canada; e.samiei@gmail.com

**Keywords:** *Cryptosporidium* detection, interdigital capacitance transducer, biosensor, label-free detection, electrodes fabrication, immobilization and surface chemistry, capacitive Immunosensor

## Abstract

*Cryptosporidium*, an intestinal protozoan pathogen, is one of the leading causes of diarrhea in healthy adults and death in children. Detection of *Cryptosporidium* oocysts has become a high priority to prevent potential outbreaks. In this paper, a label-free interdigitated-based capacitive biosensor has been introduced for the detection of *Cryptosporidium* oocysts in water samples. Specific anti-*Cryptosporidium* monoclonal antibodies (IgG3) were covalently immobilized onto interdigitated gold electrodes as the capture probes, and bovine serum albumin was used to avoid non-specific adsorption. The immobilization of the antibodies was confirmed by measuring the change in the contact angle. The detection was achieved by measuring the relative change in the capacitive/dielectric properties due to the formation of *Cryptosporidium*-antibody complex. The biosensor has been tested for different concentrations of *Cryptosporidium*. The results show that the biosensor developed can accurately distinguish different numbers of captured cells and densities on the surface of the biosensor. The number of *Cryptosporidium* oocysts captured on the electrode surface was confirmed using a fluorescein isothiocyanate (FITC) immunofluorescence assay. The response from the developed biosensor has been mainly dependent on the concentration of *Cryptosporidium* under optimized conditions. The biosensor showed a linear detection range between 15 and 153 cells/mm^2^ and a detection limit of 40 cells/mm^2^. The label-free capacitive biosensor developed has a great potential for detecting *Cryptosporidium* in environmental water samples. Furthermore, under optimized conditions, this label-free biosensor can be extended for detection of other biomarkers for biomedical and environmental analyses.

## 1. Introduction

Drinking water contamination with *Cryptosporidium* represents a major threat to human health and a significant challenge in delivering safe drinking water [1,2]. The oocyst of *Cryptosporidium* can survive and remain infective outside the host for up to 16 months, and the parasite has high resistance to the most common disinfectants [1]. *Cryptosporidium* can cause severe cryptosporidiosis, gastroenteritis in healthy adults and death in children and immuno-compromised individuals, especially patients with AIDS [3,4]. In the developing world, it is estimated that about 30% to 50% of childhood deaths are caused by *Cryptosporidium* [5]. Cryptosporidiosis is also a significant risk in the water supply for developed countries. For example, in 1993 a massive outbreak of *Cryptosporidium* in Milwaukee infected more than 400,000 people and over 100 deaths [5,6]. Additionally, *Cryptosporidium* has a significant impact on the economy both in developed and developing countries. For example, cryptosporidiosis has cost Australia and Milwaukee 45 and 96 million USD, respectively, in medical expenses [7,8]. Thus, there is a need for regular monitoring of the presence of *Cryptosporidium* in the water supply [9]. 

The presence of a low number of *Cryptosporidium* oocysts in a large volume (100 L) of water makes the detection more difficult if no concentration methods are used to increase the number of the oocysts in the sample [10]. The currently approved methods for detecting *Cryptosporidium*, such as the Environmental Protection Agency (EPA) 1623 [11], requires filtration of a large volume of water sample (minimum 10 L of water), followed by immunomagnetic separation (IMS) and staining with a fluorescence label. A fluorescence microscope is used for visualizing the labeled *Cryptosporidium*. This method is time-consuming and expensive, has limited sensitivity due to the generation of background noise and cross-reactivity [12], needs extensive sample preparation, technological improvements, and well-trained personnel [13]. Even though this method is accepted and validated for detection of *Cryptosporidium*, it is very complicated, inefficient and not suitable for on-site detection [14]. Molecular approaches for the detection of *Cryptosporidium* oocysts in water are summarized in Table 1. Hence, there is an urgent need to develop a flexible, label-free, reliable, fast and accurate detection tool to meet the challenges of detecting *Cryptosporidium* in real-time in water samples. 

A wide range of systems have been developed for water concentration and sampling directly from the water supply; however, it has been shown that they need to be coupled with one of the biological sensing layers such as in biosensing technologies to be successful [16]. Biosensors are compact analytical units used for the selective detection of chemical and biological substances using immobilized biological recognition elements on a transducer [17,18,19]. The use of biosensors has the potential to increase selectivity, sensitivity, portability, and versatility. 

Recently, there has been significant interest in label-free based detection that allows for minimizing of the labeling steps (which are time-consuming and expensive) as well as enhancing real-time detection [20,21,22]. Among different label-free methods [23], capacitive-based detection has shown many advantages including ease of detection, high sensitivity, low power consumption, size flexibility, and simplicity [24,25]. 

This paper presents the development of a multiplex label-free interdigitated capacitive based biosensor for the sensitive detection of different concentrations of *Cryptosporidium*. Compared to similar sensors reported in the literature [26], the sensor presented here does not need complicated fabrication or expensive substrates such as nanocrystalline diamond (NCD) [26] to increase sensitivity. Also, our sensing platform has shown an improvement in the efficiency and the limit of detection of *Cryptosporidium* compared to other staining and immunoassay-based detection methods [27,28]. The developed sensing platform shows great potential to be integrated into microfluidic devices or custom designed test platforms to develop a cost-effective and in-line detection device to directly test for the presence of *Cryptosporidium* oocysts in water samples. Such integration will result in the development of a portable, flexible, inexpensive, reliable and fast detection method for the early detection of *Cryptosporidium* in a real-time manner.

## 2. Materials

Purified *Cryptosporidium* parvum oocysts at the concentration of 106 oocysts/mL and FITC-labeled anti-*Cryptosporidium* parvum oocyst antibody (Crypt-a-Glo) were purchased from Waterborne, Inc., New Orleans, LA, USA. *Cryptosporidium* oocysts were kept at 4 °C in 0.1 M phosphate-buffered saline (PBS), pH 7.4. A specific antibody for the *Cryptosporidium* oocyst, i.e., IgG3 subclass monoclonal mouse, was purchased from ABD Serotec (Currently Biorad, Burlington, ON, Canada). Recombinant Protein G/thiol was purchased from protein MOD (Madison, WI, USA). Sodium phosphate monobasic monohydrate, sodium phosphate dibasic and bovine serum albumin (BSA) were purchased from Sigma (Sigma-Aldrich, St. Louis, MO, USA). All other reagents and solvents were of analytical grade, and ultrapure water was used throughout the experiments.

## 3. Experimental Methods

### 3.1. Sensor Fabrication

**Figure 1:**
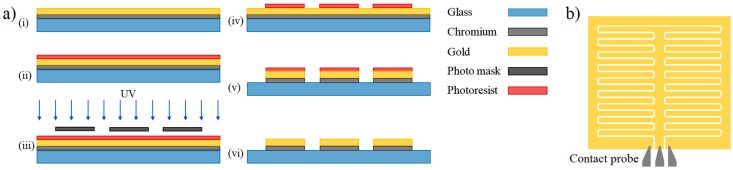
The fabricated interdigitated capacitive based biosensor. (**a**) Fabrication steps, (**b**) Layout of the IDE sensing electrodes and the schematic of the contact probe.

The fabrication of the interdigitated capacitive electrodes was completed as explained in the following steps in Figure 1. A glass slide was cleaned using piranha solution followed by 10 min oxygen plasma treatment. Chromium and gold layers were sputtered (Angstrom Engineering) in an argon atmosphere on the glass surface with the thicknesses of 50 nm and 250 nm, respectively. The electrodes were patterned using the standard lithography process and a mask with the IDE pattern [29] (Figure 1a). Each sensor has 18 pairs of the interdigitated electrodes, and each electrode has a width of 30 µm, a length of 500 µm, and a gap spacing of 30 µm (Figure 1b). Each one of the fabricated sensing electrodes works as a single biosensing platform. The interface to the electrodes is made through a coplanar waveguide probe with a pitch of 100 µm which makes contact at the location shown in Figure 1b.

### 3.2. Antibody Immobilization

**Figure 2:**
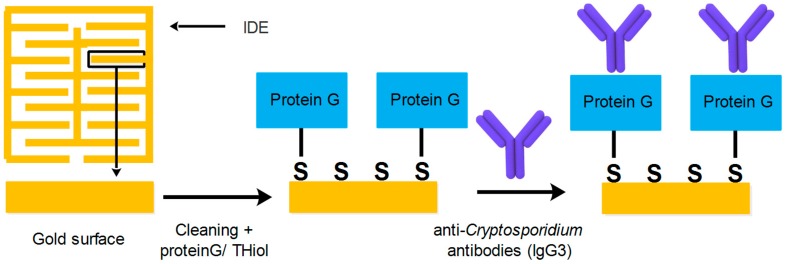
The process used for coating the surface of IDE with SAM and the immobilization of anti-*Cryptosporidium* antibodies.

The interdigitated gold electrodes were treated with plasma oxygen and were coated with a self-assembled monolayer (SAM) by adding recombinant protein G/thiol. The sensor was then incubated at 4 °C for 48 h. To wash away unbound recombinant protein G/thiol, the platform was rinsed with ultrapure water followed by phosphate-buffered saline (PBS). The sensors-SAM were then incubated with 3 µL of 100 µg/mL specific anti-*Cryptosporidium* antibodies (IgG3) at 4 °C for 24 h followed by a washing step as mentioned in the previous step. The series of the gold interdigitated electrodes were incubated with 100 mM BSA for 2 h at 4 °C to block the surface. The sensors were then washed several times with ultrapure water followed by PBS [18] (Figure 2).

### 3.3. Electrochemical Measurements

The formation of SAM and antibodies immobilization on gold IDE was checked using an electrochemical cell equipped with a three-electrode system. The fabricated gold IDE was used as the working electrode. An external platinum wire and an Ag/AgCl/3.0 M KCl electrode were used as the counter and the reference electrodes, respectively. A salt bridge, filled with 1 M KNO3 aqueous solution and agar, was used to reduce chloride ion diffusion into the electrolyte solution. Cyclic voltammetry (CV) was recorded for the bare gold, SAM, and antibodies using a VersaSTAT 4 potentiostat (Princeton Applied Research) in a potential range from −300 to +500 mV (against Ag/AgCl) at a scanning rate of 50 mV/s. The electrolyte used for the CV measurement consisted of a 5-mM K_4_Fe(CN)_6/5_ mM K_3_Fe(CN)_6_ aqueous solution with 1 M NaClO_4_ as the supporting electrolyte. An open circuit potential was used in all electrochemical measurements to be consistent. 

All fabricated working electrodes were cleaned prior to making measurements by incubation for 30 s in piranha solution (H_2_SO_4_-H_2_O_2_ 3:1 (*v*/*v*)), rinsed with ultrapure water, and then polished with different sizes of alumina slurry (0.1 mm and 0.05 mm, respectively) for 2 min for each size. All electrodes were further cleaned electrochemically by cycling them in a 0.5-M KOH (basic) solution in the range of −2 to 0 V versus the reference electrode (Ag/AgCl) followed by cycling them in a 0.5 M H_2_SO_4_ (acidic) solution in the range of 0 to +1.5 V.

### 3.4. Contact Angle Measurements

Contact angles were determined to further verify the immobilization of the antibodies on the surface. The measurement was performed similarly to the method explained in [18] showing that the presence of the antibodies on the surface changes the surface properties of the sensor from hydrophilic to superhydrophilic [18]. Two substrates were prepared: one with the chromium and gold layers, and the other with the immobilized antibodies on the chromium and gold layers. A water droplet was dispensed on the surface of the substrates using a syringe pump. The water was dispensed through a needle with a hydrophobic outer surface. The surface of the needle was coated with a hydrophobic material to avoid the effect of the needle on the contact angle on the surface. The contact angle was measured from the side view images taken in the process of the experiment [18].

### 3.5. Sample Preparation and Measurement

**Figure 3:**
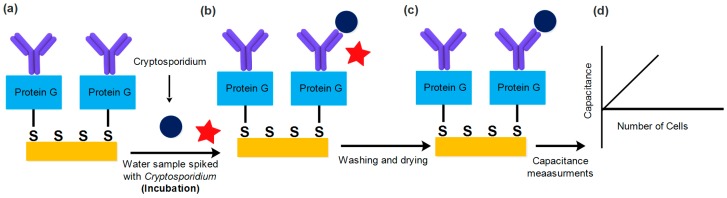
Diagram illustrating the detection process: (**a**) incubation of the anti-*Cryptosporidium* antibodies by coating the sensing electrodes with water samples containing different concentrations of *Cryptosporidium*; (**b**) washing with phosphate-buffered saline (PBS) of pH 7 to remove any unbound and non-specific molecules; (**c**) drying the interdigitated electrodes; and (**d**) capacitance measurements over a specific frequency range.

Series of dilutions of *Cryptosporidium* samples were prepared using phosphate-buffered saline (PBS) as the diluent. Typical dilutions were 250, 200, 150, 100, 50, and 0 oocysts in a final volume of 5 µL. Different concentrations in the final volume of 5 µL were incubated for 20 min at room temperature (23 °C) with the sensing electrodes functionalized with specific anti-*Cryptosporidium* antibodies (ABD Serotec, Burlington, ND Canada) (Currently Biorad). All electrodes were washed with PBS, then with ultrapure water to remove any unbound and loosely bound *Cryptosporidium* from the electrode surface (Figure 3). The samples were dried in air for 30 min, and measurements were performed. Capacitance measurements were obtained before and after the incubation of *Cryptosporidium* using a network analyzer (N5241A, Agilent Technologies, Santa Clara, CA, USA) connected to a coplanar waveguide probe which makes a calibrated impedance measurement referenced to the contact interface of the electrodes. S-parameter measurements [30] were performed ver a frequency range of 5.5 GHz to 8.5 GHz and converted to equivalent circuit capacitance values. The network analyzer was calibrated using the SOLT (short-open-load-through) method, and the S-parameter values were recorded at two different stages: (1) after SAM formation with immobilized anti-*Cryptosporidium* antibodies, and (2) after incubating the sensor with various concentrations of *Cryptosporidium*. All measurements were repeated three times to conduct a statistical error analysis and to confirm the detection limit. The changes in the capacitance and dielectric properties between negative control and after capturing *Cryptosporidium* were compared. 

The following equation was used to calculate the relative capacitance for each concentration (from the generated data) as a function of the frequency for four different cell densities ranging from 15 to 152 cells/mm^2^ of the captured *Cryptosporidium* oocysts on the sensing surface.

(1) $$\%\;\Delta C\;=\;\frac{C-C_{0}}{C_{0}}\;\times 100$$

In the above relation, $C_{o}$ is the capacitance before binding of *Cryptosporidium* to the immobilized antibodies, and C is the capacitance after binding of a specific concentration of *Cryptosporidium*.

### 3.6. FITC Measurements

The number of *Cryptosporidium* oocysts captured on the electrode surface was confirmed using an immunofluorescence assay. Staining was carried out according to the manufacturer: (a) all electrodes were stained with fluorescein isothiocyanate (FTIC)-conjugated anti-*Cryptosporidium* oocysts monoclonal antibodies (mAb) (Crypto-a-Glo kit, Waterborne, Inc, New Orleans, LA, USA); (b) all electrodes were incubated in a humid chamber at room temperature for 30 min; (c) the electrodes were rinsed by adding the 50–100 µL washing buffer and left for 1 min at room temperature (23 °C); (d) the electrode surfaces were tilted slightly to remove the washing buffer; and (e) an absorbent material was placed at the edge of the electrodes to absorb the excess fluid on the surface. Electrodes were imaged with a 20× epifluorescence microscope (Zeiss AxioVision, Thornwood, NY, USA), and the number of oocysts was counted.

## 4. Results and Discussion

**Figure 4:**
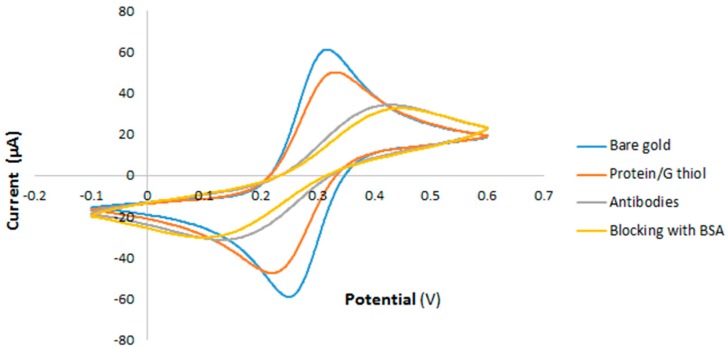
Cyclic voltammetry (CV) for the case of bare Au electrode (blue curve), added protein/thiol (red curve), immobilized anti-*Cryptosporidium* antibodies (gray curve), and added bovine serum albumin (BSA) (yellow curve).

Cyclic voltammetry (CV) and contact angle measurement were conducted step by step to confirm the formation of the SAM and the immobilization of the antibodies. The CV measurements for the different stages of the electrode modification with protein/thiol and antibodies are shown in Figure 4.

A redox peak pair was observed for the bare IDE (blue curve). The current decreased after modifying the electrodes with protein/thiol (red curve), after immobilization of antibodies (gray curve), and after blocking with BSA (yellow curve). The generated results from the CV analysis confirmed the formation of SAM, immobilization of the antibodies and the surface blocking step. Functionalization of the surface by antibodies was also confirmed by contact angle measurement. For this purpose, advancing and receding contact angles were measured which shows the level of wettability of the surface. In essence, immobilization of biological species on the surface significantly increases the wettability of the surface [18]. Advancing and receding contact angles were measured as 90° and 46° (±1°) for the gold surface, respectively, and 57° and 4° for the gold surface with immobilized antibodies, respectively. The measured contact angles show the gold surface has hydrophilic properties with low receding contact angles, while the surface functionalized with antibodies is superhydrophilic with an extremely small receding contact angle.

**Figure 5:**
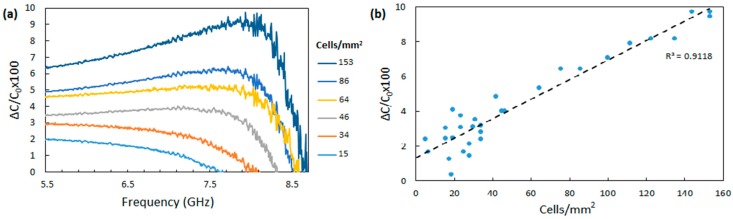
Results of the relative capacitance measurement for various concentrations of *Cryptosporidium*. (**a**) Relative capacitance change measurements for different concentrations of *Cryptosporidium* for samples ranging from 15 cells/mm^2^ to 153 cells/mm^2^. (**b**) The calibration curve generated from capacitance measurements.

Capacitance measurements were obtained first for the modified IDE with the immobilized anti-*Cryptosporidium* antibodies ($C_{o}$) and then after capturing the *Cryptosporidium* oocysts (C). These values are correlated to the number of the *Cryptosporidium* oocysts bound to the immobilized antibodies. Figure 5 shows the change in the capacitance values (ΔC/$C_{o}$%) as a function of the frequency for six different cell densities (from 15 to 152 cells/mm^2^) of captured *Cryptosporidium* oocysts on the sensing surface (Figure 5a). A distinct change in capacitance was measured for different concentrations of *Cryptosporidium* over the frequency range of 5.5 GHz to 8.5 GHz. The statistical error, average values, and the lowest detection limit were calculated. The results are shown in Figure 5a and an increase in the measured relative capacitance (ΔC) as a function of *Cryptosporidium* concentration is observed. The maximum change in the response due to the presence of different cell densities was observed at 7.5 GHz (Figure 5a). Hence, for each number of the captured oocysts, the maximum value of the relative change in the capacitance at 7.5 GHz was used to generate the calibration curve (Figure 5b). The curve is linear over a range of 15 to 153 cells/mm^2^, showing the effectiveness of the platform as a cost-effective, label-free and sensitive tool. This curve also shows the detection limit is approximately 40 cells/mm^2^ and, below this limit, the measurement error increases significantly. 

**Figure 6:**
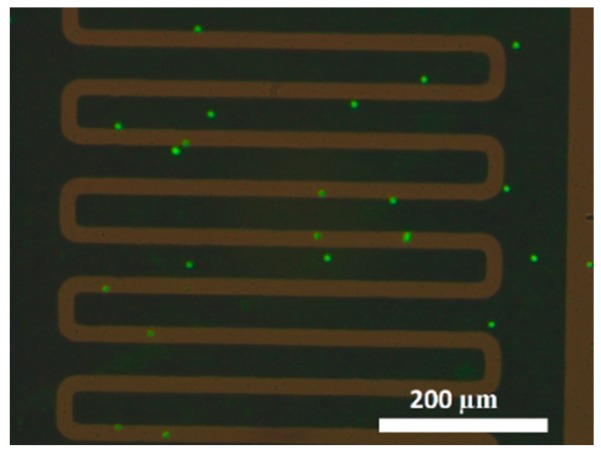
Fluorescence microscopic images illustrate 20 cells of *Cryptosporidium* bound to the immobilized antibodies on a 1-mm^2^ electrode surface area using direct fluorescein isothiocyanate (FITC)-conjugated anti-*Cryptosporidium* oocysts monoclonal antibodies.

After the capacitance measurement step, specific immunofluorescence assays for *Cryptosporidium* were used to confirm the number of *Cryptosporidium* oocyst captured and detected by the developed sensor as explained in Section 3.5. Fluorescence microscopic images were taken, and the number of *Cryptosporidium* bound to the antibodies immobilized onto the IDE surface were counted and correlated to the capacitance measurements. The fluorescence results confirm the selectivity of the sensor, with anti-*Cryptosporidium* antibodies on the surface, towards *Cryptosporidium* (Figure 6).

## 5. Conclusions

This paper reported a label-free interdigitated capacitive based biosensor for the detection of *Cryptosporidium*. The sensitivity and detection limits of the sensing platform were measured. The results showed a linear detection range for *Cryptosporidium* concentrations with a detection limit of 40 cells/mm^2^. The developed sensing platform demonstrates the potential of implementing a biosensing platform which can reduce the need for trained technicians and specialized laboratories. Furthermore, sample pretreatments such as sonication and UV are not required while using this platform [31,32,33], which has high flexibility. In the future, the developed sensing platform will be tested with real samples to confirm the applicability of the technology as a replacement for the staining and the fluorescence detection part in the EPA 1623 method. The label-free biosensor developed in this research can be extended for detection of other biomarkers in a variety of biomedical and environmental analyses through the emerging biosensing technology. This technology also has a great potential to be integrated into microfluidic devices for portable, personalized and point-of-care (POC) applications.

## Figures and Tables

**Table 1:** Molecular approaches to detect *Cryptosporidium* oocysts in water [15].

Technique	Filtration & Capacity	Concentration	Purification	Detection	Recovery (%)
ASTM, 1991, USEPA, 1996 (USA)	Cartridge filtration (1.0 µm) 100–1000 L	Centrifuged 1050× *g* 10 min	Percoll-sucrose density-gradient centrifuging	IFA, DIC microscopy	0–100
Method 1622/1623: USEPA, 1999a, USEPA, 1999b (USA)	Membrane filter (Envirochek™ HV) 10–1000 L	Centrifuged 1100× *g* 15 min	Dynal IMS		12–93 (21–100)
SOP 1999, SI No. 1524 ¥ (UK)	Genera filta-Max™ filter membranes		Dynal IMS	IFA, DIC microscopy	30–50
